# Familial dilated cardiomyopathy in a child: a case report

**DOI:** 10.1186/s12887-024-04614-4

**Published:** 2024-04-01

**Authors:** Ali Ismail, Dima Khreis, Amani Assaad, Marianne Nimah Majdalani

**Affiliations:** Department of Pediatric and Adolescent Medicine, American University of Beirut Medical Center, PO Box: 11-0236. Riad El Solh, Beirut, Beirut, 1107 2020 Lebanon

**Keywords:** Dilated cardiomyopathy, Cardiogenic shock, Pediatrics, Echocardiogram, Genetics

## Abstract

**Background:**

Dilated cardiomyopathy (DCM) commonly leads to heart failure (HF) and represents the most common indication for cardiac transplantation in the pediatric population. Clinical manifestations of DCM are mainly the symptoms of heart failure; it is diagnosed by EKG, chest x-ray and echocardiography. For the idiopathic and familial diseases cases of DCM, there are no definite guidelines for treatment in children as they are treated for prognostic improvement.

**Case presentation:**

We report the case of a 2-year-old girl diagnosed with dilated cardiomyopathy associated with homozygous mutation in the Myosin Light Chain 3 gene admitted for edema in lower extremities, muscle weakness, lethargy and vomiting, and she was found to be in cardiogenic shock. Chest x-ray showed cardiomegaly and EKG showed first degree atrioventricular block. Echocardiogram showed severe biventricular systolic and diastolic dysfunction. After 70 days of hospitalization, the patient went into cardiac arrest with cessation of electrical and mechanical activity of the heart, despite cardiopulmonary resuscitative efforts.

**Conclusion:**

Although rare, pediatric DCM carries a high risk of morbidity and mortality and a lack of curative therapy.

## Introduction

Dilated cardiomyopathy (DCM) is a myocardial disorder leading to left ventricular or biventricular dilation and impaired contraction, with no underlying cardiovascular cause (coronary artery disease, hypertension, valvular disease, or congenital heart disease) [1].

Although rare in children [2], DCM is a debilitating heart disease often leading to heart failure. Despite advances in medical treatment, it represents the main indication for heart transplantation globally. Approximately 40% of children with DCM require heart transplantation or pass away within 2 years of diagnosis [3].

Pediatric cardiomyopathies have a wide variety of causes, ranging from genetic mutations, infections, inflammation, and auto-immune diseases to exposure to toxins and endocrine or neuromuscular causes [4]. When no obvious etiology is found, cardiomyopathy affecting a single family member is classified as *idiopathic*. On the other hand, *familial* dilated cardiomyopathy (FDCM) diagnosis can be made in the presence of two or more family members with cardiomyopathy with no apparent etiology, or after the sudden death of a first-degree relative of a patient with DCM before the age of 35 years [5].

Idiopathic and familial diseases are the most reported forms of DCM. In most cases FDCM is inherited as an autosomal dominant pattern (30%–50%), while X-linked, autosomal recessive and mitochondrial inheritance patterns are less common. Ongoing advances in genetic sequencing and molecular methods revealed more than 35 gene-mutations reported to cause familial cardiomyopathy (FDC) [6].

Though management of pediatric heart failure has advanced, children with FDCM lack definite guidelines for therapy as most treatment protocols for children are based on those used for adults [7]. In this paper we describe the case of a 2-year-old girl with FDCM with homozygous mutation in the MYL3 gene.

## Case presentation

**Fig. 1:**
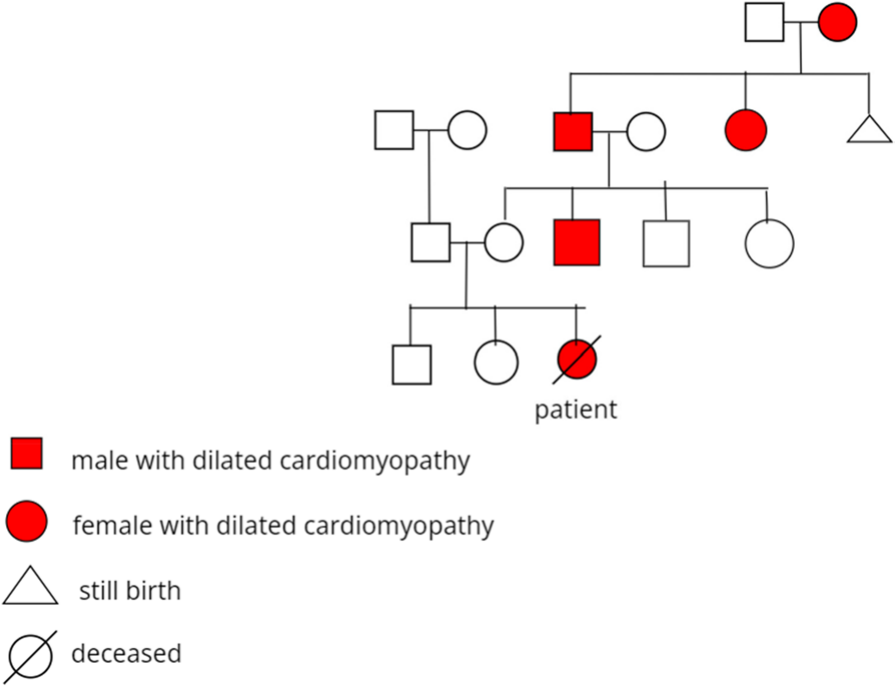
Pedigree of familial dilated cardiomyopathy. Our patient is indicated in the figure above

A 2-year-old girl, with a family history of early-onset cardiomyopathy (Fig. 1) and a medical history of recurrent upper respiratory tract infections necessitating only symptomatic care, visited her primary care pediatrician with symptoms of increasing lethargy, cough, shortness of breath after minimal exercise (such as walking, eating, and crying), puffy eyes, and edema in the lower extremities. Subsequently, she was referred to a pediatric cardiologist, who conducted an echocardiogram and baseline laboratory tests in a private clinic setting. The initial assessment revealed heart failure with a 30% ejection fraction. Following this diagnosis, the patient received outpatient treatment consisting of oral medications (Furosemide, Captopril, Spironolactone, Digoxin, and Carvedilol) and underwent frequent follow-up visits.

During the 7-month period following the initial diagnosis, the patient's condition remained stable with no significant improvement or deterioration until one week prior to her presentation at our Emergency Department of the American University of Beirut Medical Center (AUBMC). At that time, she experienced frequent vomiting, worsening lethargy, and increased shortness of breath leading to a state of cardiogenic shock.

On presentation, she was ill-looking with mottled skin, cyanotic lips, cold extremities, weak peripheral pulses and delayed capillary refill time. She was afebrile, tachypneic, tachycardic, and hypotensive (respiratory rate (RR) of 60 breaths/minute, heart rate (HR) of 166 beats/minute, blood pressure (BP) of 78/31 mmHg and percutaneous oxygen saturation (SpO2) of 100% on face mask oxygen 5 liters/minute). Auscultation revealed faint heart sounds and diffuse rhonchi over the chest. Mild hepatomegaly was also noted.

**Fig. 2:**
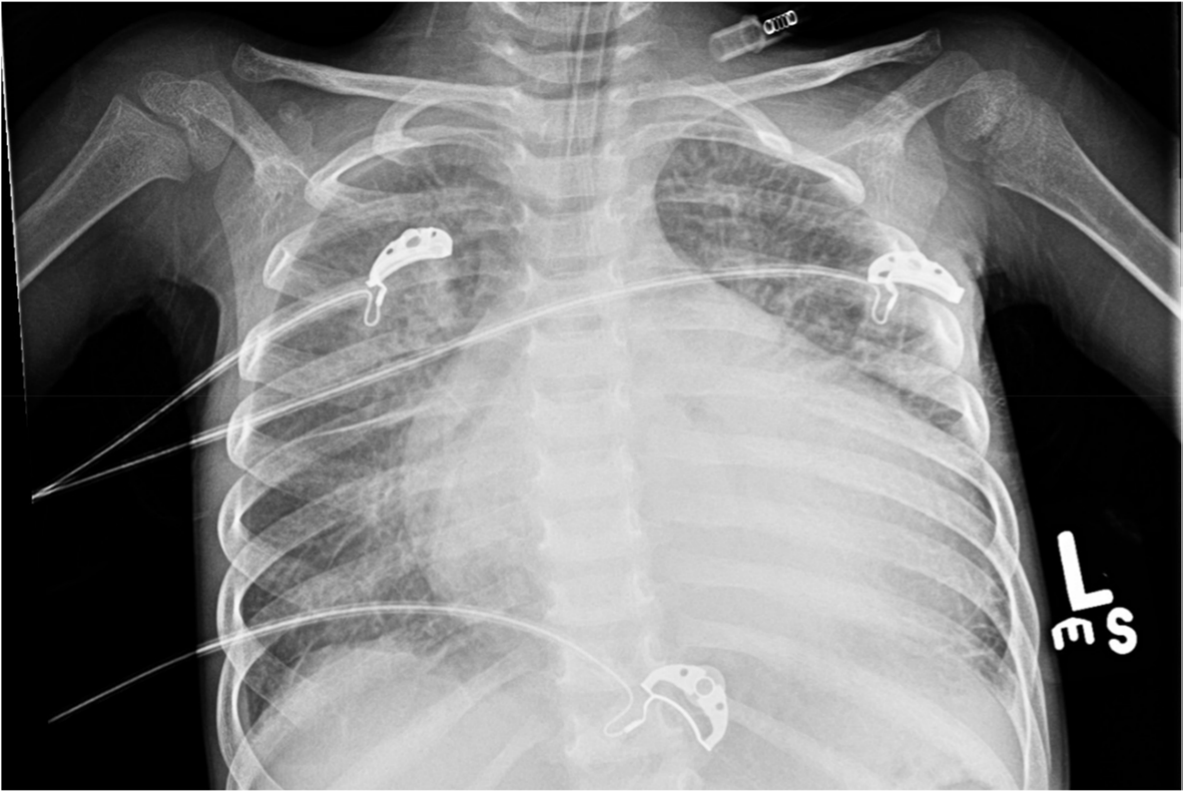
Chest X-Ray upon admission: The cardiac silhouette is significantly enlarged. There are increased interstitial markings with cephalization of the pulmonary vessels and Kerley B lines in keeping with interstitial pulmonary edema. There is blunting of both costophrenic angles suggestive of small bilateral pleural effusions. No pneumothorax. Increased retrocardiac opacity silhouetting the medial aspect of the left hemidiaphragm, atelectatic or infectious in nature

**Fig. 3:**
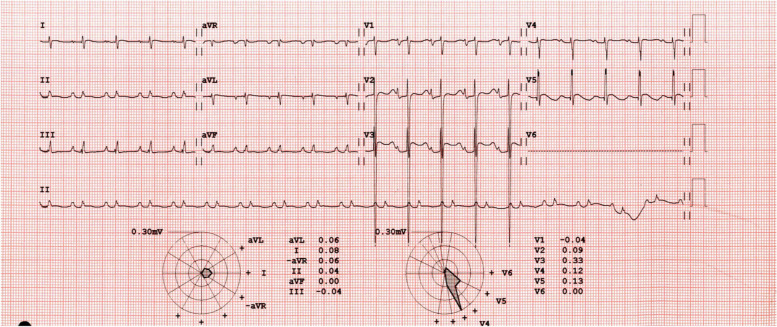
EKG for our patient upon admission showing first degree heart block

**Fig. 4:**
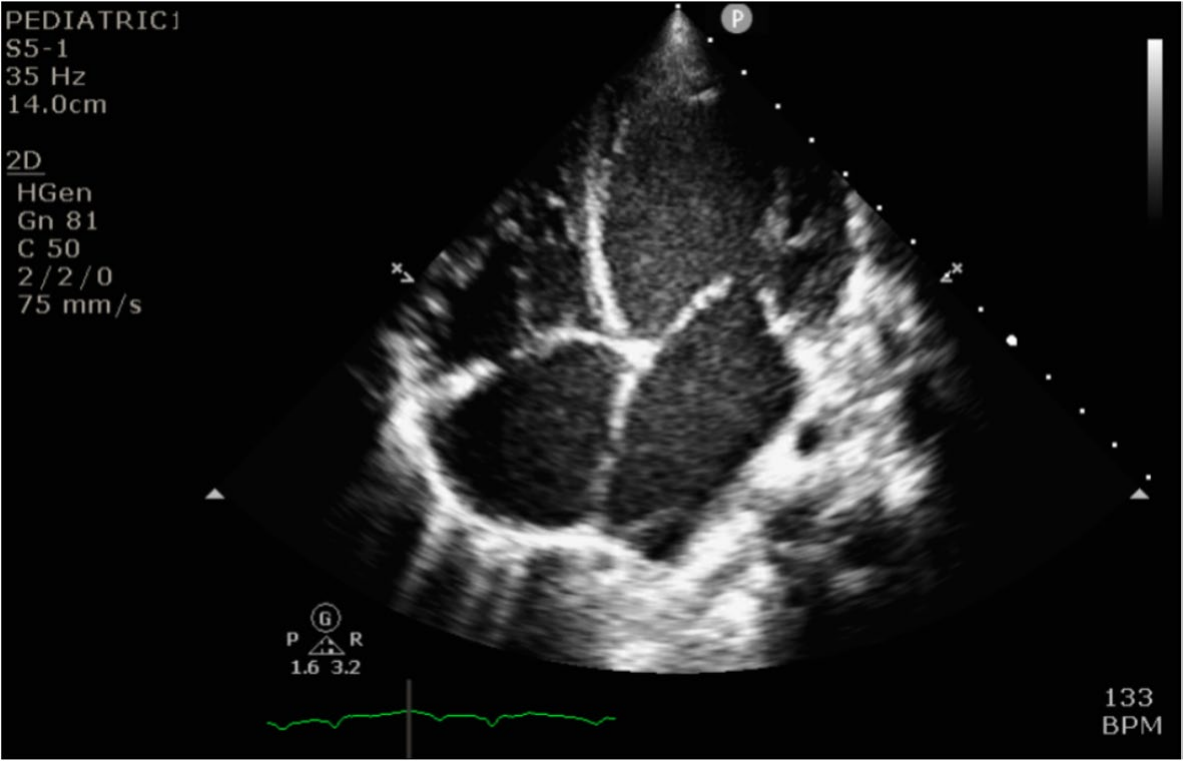
Echocardiogram upon admission showing dilated left ventricle

Initial laboratory assessment (Table 1) was consistent with severe metabolic and respiratory acidosis as shown by arterial blood gases (PH: 7.01; PCO2: 51 mmHg; Bicarbonate: 13 mmol/L; Base excess: -18 mmol/L; lactic acid: 9.8 mmol/L). Chest X-Ray revealed cardiomegaly with increased interstitial marking (Fig. 2) and EKG revealed first degree atrioventricular block (Fig. 3). Echocardiogram on arrival showed severe biventricular systolic and diastolic dysfunction (ejection fraction less than 20%), with moderate bilateral atrioventricular valve regurgitation, left ventricular outflow tract velocity time integral (LVOT VTI) of 4 (Fig. 4). Evidence of left ventricle diastolic dysfunction included a Mitral E/A ratio of 1.9, an E wave deceleration time of 35 msec, elevated systolic pressure in the pulmonary artery, PV A wave reversal exceeding the duration of the MV A wave, along with a severely dilated left atrium. A diagnosis of decompensated dilated cardiomyopathy with cardiogenic shock was made.

**Table 1:**
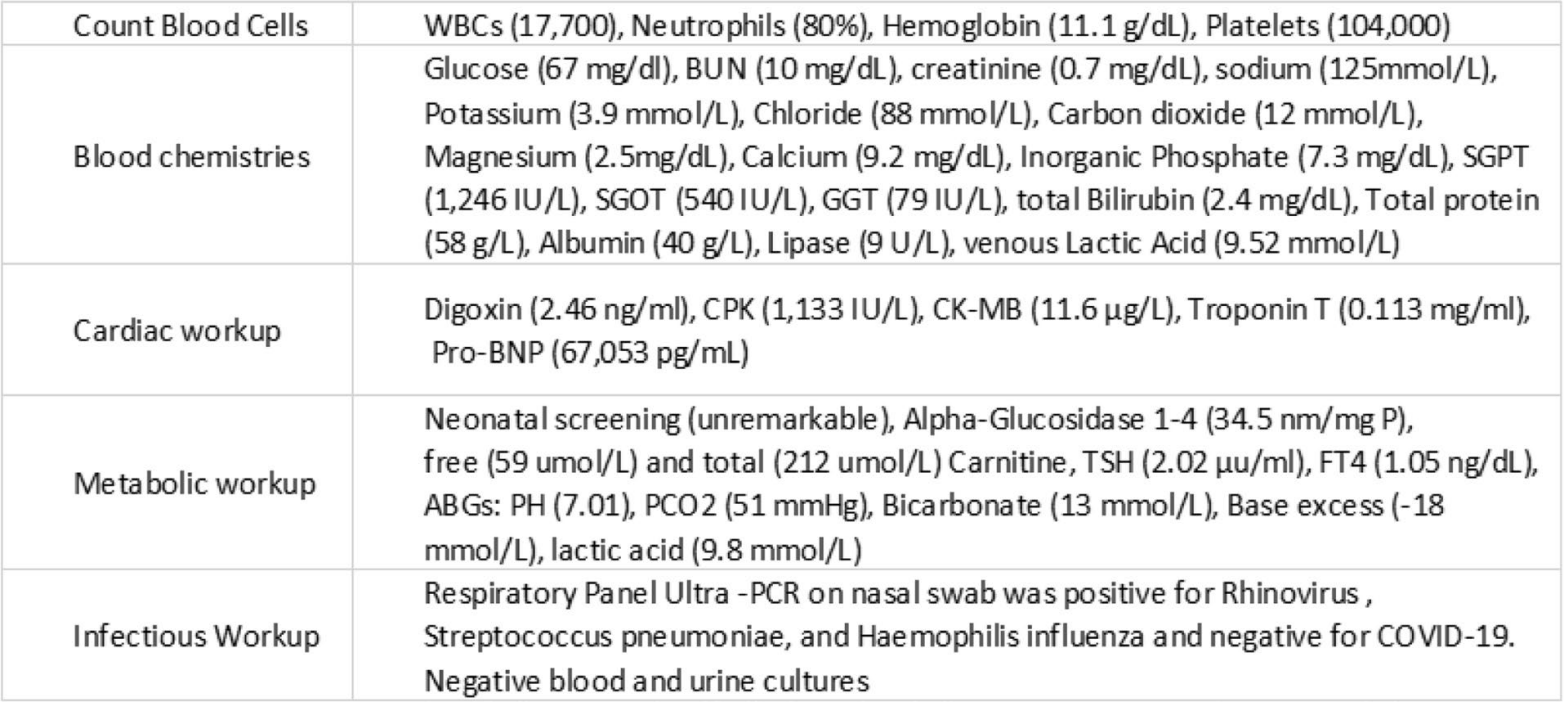
Initial laboratory findings

Patient was intubated and started on Epinephrine drip, Milrinone drip and diuretics. She was admitted to Pediatric Intensive Care Unit (PICU) for extensive diagnostic workup and management where she received an advanced multidisciplinary care including cardiology, metabolic diseases, critical care, infectious diseases and nephrology specialists.

She remained intubated and mechanically ventilated throughout her hospital stay. Serial echocardiographic assessments revealed worsening ventricular function (EF less than 17%), despite various cardiac medications (Epinephrine, Norepinephrine, Milrinone, Dobutamine, Digoxin, Carvedilol), and aggressive diuresis (Furosemide, Spironolactone, Acetazolamide, Hydrochlorothiazide, Bumetanide). Two doses of intravenous Levosimendan infusions were also administered; the third dose was stopped due to transient hypotension.

Metabolic and genetic work up was done and was negative. Whole exome sequencing later revealed a homozygous mutation in the MYL3 (Myosin Light Chain 3) gene, associated with familial autosomal dominant MYL3-related cardiomyopathy.

Her hospital stay was complicated by multiple infections including multilobar pneumonia and candiduria, for which she received broad spectrum antibiotics and antifungals. Progressively, the child’s condition continued to deteriorate as she developed end-organ dysfunction, including renal and hepatic failure with severe lactic acidosis and disseminated intravascular coagulation. After a total of 70 days of hospitalization, the patient developed rare ectopic beats then went into cardiac arrest followed by a cessation of electrical and mechanical activity of the heart, despite cardiopulmonary resuscitative efforts.

## Discussion

FDCM, which represents around 20-35% of DCM cases [8], is a significant health concern in the pediatric population, with high rates of decompensated heart failure often requiring heart transplantation and leading to early cardiac death [3].

Over the past 20 years, significant progress has been achieved in understanding the genetics of FDCM, with over 50 associated genes recognized so far [9–11]. Most genes, which encode proteins of the sarcomere of the cardiomyocytes, are transmitted in an autosomal dominant fashion [6]. Our patient was found to have FDCM based on the presence of DCM in multiple relatives. A homozygous variant was later identified by whole exome sequencing in the MYL3 gene, which encodes the ventricular myosin essential light chain (ELC) [12]. The identified MYL3 variant c.560-2A>G is predicted to disrupt the highly conserved acceptor splice site of the last exon of this gene. Pathogenic variants in this gene are associated with autosomal dominant MYL3-related cardiomyopathy, particularly familial hypertrophic cardiomyopathy. Additionally, heterozygous and homozygous variants in the MYL3 gene have been reported in patients with dilated cardiomyopathy, including loss-of-function variants [13]. Nevertheless, based on current evidence, the clinical relationship of this variant with dilated cardiomyopathy is still unclear. Therefore, the gene is reported as disputed and its variant c.560-2A>G is classified as a variant of uncertain significance (class 3) according to the recommendations of the American College of Medical Genetics and Genomics (ACMG) [14]. In our case, parental targeted genetic testing was recommended to confirm the inheritance of MYL3 gene variant and to establish whether this gene variant is correlated with FDCM. However, the parents refused to test themselves because of the high cost of genetic testing in our country.

In most cases, patients with DCM present with symptoms related to heart failure [15]. The degree of severity might range from mild to severe including shock, cardiac arrhythmias, thromboembolic complications, and sudden cardiac death at any stage of the disease. In our patient, symptoms of heart failure were the reason for admission but then progressed to multiorgan failure followed by sudden cardiac arrest after 70 days of hospitalization.

Regarding diagnosis, DCM is diagnosed using EKG, chest x-ray and echocardiography. Despite progression in the genetics of FDCM, there has not been a corresponding improvement in the development of targeted clinical management [16]. And in spite of the poor prognosis, it has been demonstrated that current therapeutic approaches can lead to prognostic improvement [17].

Generally, neurohormonal antagonists, angiotensin converting enzyme inhibitors (ACEi) or angiotensin II type I receptor blockers (ARBs) in association with beta-blocker are recommended as first line treatment. The addition of mineralocorticoid receptor antagonists (MRA) should be considered in patients who fail to respond to treatment with ACEi/ARBs and beta-blocker [18, 19]. To note that the majority of treatment options were extrapolated from adult studies and need to be further studied in the pediatric population [20].

Levosimendan is a calcium sensitizer renowned for its favorable inotropic effects, anti-inflammatory, anti-oxidant, and possibly cardioprotective properties [21–24]. Our patient received 3 doses of Levosimendan. After the first 2 doses, improvement was noted however, transient hypotension developed after the third dose for which the medication was subsequently stopped. Despite concerns about its cost effectiveness and adverse effects such as hypotension, headache, atrial fibrillation, hypokalemia and tachycardia [25], repetitive infusions of Levosimendan in children with severe DCM appeared to be hemodynamically well tolerated [24–26]. While repetitive infusions of Levosimendan in children with advanced heart failure might be beneficial, the optimal dosing and intervals have not yet been concluded [27].

Multiple studies have also shown that supplementation of Levocarnitine, an amino acid-derived molecule, boosts energy metabolism in cardiomyocytes by promoting myocardial fatty acid oxidation [28, 29]. Cardioprotective effects of L-carnitine also include inhibition of cardiac fibrosis, nitric oxide generation, and interstitial myocardial remodeling [30]. Our patient received Levocarnitine supplementation throughout her hospital stay, despite no detected carnitine deficiency.

Finding alternate therapy options for children with DCM and heart failure symptoms refractory to medical treatment has received a great deal of focus during the past 20 years. Novel pacing strategies and mechanical assist devices were implemented as a bridge for heart transplantation or functional recovery [31].

Pediatric heart transplant represents an important treatment option for end-stage heart disease in the past few decades, particularly in children with DCM in heart failure. Since the first heart transplantation in an infant in 1967 [32], post-transplantation outcomes have significantly evolved due to advances in surgical technique and immunosuppressive therapy, with 1-year survival rates approaching 90% [33]. Despite that, the Middle East region lacks successful stories of heart transplantation due to many challenges, including: ethical issues and lack of awareness about organ donation and transplantation leading to shortage of donors, unavailability of advanced cardiac centers with expert pediatric cardiothoracic surgeons and post-transplant multidisciplinary care teams, as well as excessive costs of such procedure and the burden of procurement of novel immunosuppressive therapy [34–36].

## Conclusion

Dilated cardiomyopathy is the number one indication for heart transplantation in children. Idiopathic and familial diseases are the most common reported causes of DCM. Clinical presentation includes symptoms of heart failure and diagnosis is usually made by EKG, chest x-ray and echocardiography. In accordance with the literature, over 50 associated genes were recognized so far related to the disease [9–11]. Despite this, there has been no improvement in development of gene targeted management. Regarding our patient, there were no reported cases in the literature mentioning the MYL3 variant c.560-2A>G. Therefore, genetic screening is important when familial DCM is suspected in order to compare different variants related to DCM and to develop targeted treatment.

## Data Availability

Data sharing is not applicable to this article as no datasets were generated or analyzed during the current study.

## References

[1] Schultheiss HP (2019). Dilated cardiomyopathy. Nat Rev Dis Primers.

[2] Towbin JA, Lowe AM, Colan SD, Sleeper LA, Orav EJ, Clunie S, Messere J, Cox GF, Lurie PR, Hsu D, Canter C, Wilkinson JD, Lipshultz SE (2006). Incidence, causes, and outcomes of dilated cardiomyopathy in children. JAMA..

[3] Lipshultz SE (2013). Pediatric cardiomyopathies: causes, epidemiology, clinical course, preventive strategies and therapies. Future Cardiol.

[4] Lipshultz SE (2019). Cardiomyopathy in Children: Classification and Diagnosis: A Scientific Statement From the American Heart Association. Circulation..

[5] Hershberger RE, Siegfried JD (2011). Update 2011: clinical and genetic issues in familial dilated cardiomyopathy. J Am Coll Cardiol.

[6] Park MK et al. Park’s Pediatric Cardiology For Practitioners, seventh edition, 2020- p. 255-257.

[7] Angelis De (2020). Cardiomyopathies in children: classification, diagnosis and treatment. Curr Opinion Organ Transpl.

[8] Burkett EL, Hershberger RE (2005). Clinical and genetic issues in familial dilated cardiomyopathy. J Am College Cardiol.

[9] Hershberger R, Hedges D, Morales A (2013). Dilated cardiomyopathy: the complexity of a diverse genetic architecture. Nat Rev Cardiol.

[10] Van Spaendonck-Zwarts KY, van Rijsingen IAW, van den Berg MP (2013). Genetic analysis in 418 index patients with idiopathic dilated cardiomyopathy: overview of 10 years’ experience. Eur J Heart Fail.

[11] Millat G, Bouvagnet P, Chevalier P (2011). Clinical and mutational spectrum in a cohort of 105 unrelated patients with dilated cardiomyopathy. Eur J Med Genet.

[12] Zhao Y, Feng Y, Zhang YM, Ding XX, Song YZ, Zhang AM, Liu L, Zhang H, Ding JH, Xia XS (2015). Targeted next-generation sequencing of candidate genes reveals novel mutations in patients with dilated cardiomyopathy. Int J Mol Med.

[13] Osborn D, Emrahi L, Clayton J, Tabrizi MT, Wan A, Maroofian R, Yazdchi M, Garcia M, Galehdari H, Hesse C, Shariati G, Mazaheri N, Sedaghat A, Goullée H, Laing N, Jamshidi Y, Tajsharghi H (2021). Autosomal recessive cardiomyopathy and sudden cardiac death associated with variants in MYL3. Genet Med.

[14] Jordan E, Peterson L, Ai T, Asatryan B, Bronicki L, Brown E, Celeghin R, Edwards M, Fan J, Ingles J, James CA, Jarinova O, Johnson R, Judge DP, Lahrouchi N, Lekanne Deprez RH, Lumbers RT, Mazzarotto F, Medeiros Domingo A, Miller RL, … Hershberger RE. (2021). Evidence-Based Assessment of Genes in Dilated Cardiomyopathy. Circulation. 144(1):7–19. 10.1161/CIRCULATIONAHA.120.053033 10.1161/CIRCULATIONAHA.120.053033 PMC8247549 33947203

[15] Mahmaljy H, Yelamanchili VS, Singhal M. (2021). Dilated cardiomyopathy. In StatPearls. StatPearls Publishing. 28722940

[16] Magliola R, Moreno G, Vassallo JC (2009). Levosimendan, a new inotropic drug: experience in children with acute heart failure. Arch Argent Pediatr.

[17] Rizos. Three-year survival of patients with heart failure caused by dilated cardiomyopathy and L-carnitine administration. Am Heart J. 2000;139(2):s120–s123. 10.1067/mhj.2000.103917 10650325

[18] Ponikowski P, Voors AA, Anker SD, Bueno H, Cleland JGF, Coats AJS (2016). 2016 ESC guide- lines for the diagnosis and treatment of acute and chronic heart failure: the task force for the diagnosis and treatment of acute and chronic heart failure of the European Society of Cardiology (ESC) developed with the special contribution of the Heart Failure Association (HFA) of the ESC. Eur Heart J.

[19] Kushwah S, Kumar A, Sahana KS (2016). Levosimendan. A promising future drug for refractory cardiac failure in children?. Indian Heart J.

[20] Silva JN, Canter CE (2010). Current management of pediatric dilated cardiomyopathy. Curr Opin Cardiol..

[21] Papp Z, Édes I, Fruhwald S (2012). Levosimendan: molecular mechanisms and clinical implications: consensus of experts on the mechanisms of action of levosimendan. Int J Cardiol.

[22] P.M. Janssen, N. Datz, O. Zeitz, G. Hasenfuss Levosimendan improves diastolic and systolic function in failing human myocardium. Eur J Pharmacol. 2000;404:191-199. 10.1016/s0014-2999(00)00609-9 10980279

[23] Parissis JT, Adamopoulos S, Antoniades C (2004). Effects of levosimendan on circulating pro-inflammatory cytokines and soluble apoptosis mediators in patients with decompensated advanced heart failure. Am J Cardiol..

[24] Suominen P, Mattila N, Nyblom O, Rautiainen P, Turanlahti M, Rahkonen O (2017). The hemodynamic effects and safety of repetitive levosimendan infusions on children with dilated cardiomyopathy. World J Pediatr Congenital Heart Surg.

[25] Nieminen MS, Fruhwald S, Heunks LM, Suominen PK, Gordon AC, Kivikko M, Pollesello P (2013). Levosimendan: current data, clinical use and future development. Heart Lung Vessels.

[26] Simona S (2015). A systematic review on levosimendan in paediatric patients. Curr Vasc Pharmacol.

[27] Figgitt DP, Gillies PS, Goa KL (2001). Levosimendan. Drugs.

[28] Wang Y, Xu Y, Zou R (2018). Effect of levocarnitine on the therapeutic efficacy of conventional therapy in children with dilated cardiomyopathy: results of a randomized trial in 29 children. Paediatr Drugs.

[29] Weng Y (2021). Efficacy of L-Carnitine for Dilated Cardiomyopathy: A Meta-Analysis of Randomized Controlled Trials. BioMed Res Int.

[30] Flanagan JL, Simmons PA, Vehige J, Willcox MD, Garrett Q (2010). Role of carnitine in disease. Nutr Metab (Lond).

[31] Cohen JA, Almodovar MC (2020). Dilated cardiomyopathy in children: moving beyond traditional pharmacologic therapy. Curr Opinion Cardiol.

[32] Kantrowitz A, Haller JD, Joos H (1968). Transplantation of the heart in an infant and an adult. Am J Cardiol.

[33] Tsirka E, Trinkaus K, Chen S (2004). Improved outcomes of pediatric dilated cardiomyopathy with utilization of heart transplantation. J Am Coll Cardiol..

[34] Bader F, Atallah B, Rizk J, AlHabeeb W (2018). Heart Transplantation in the Middle East Gulf Region. Transplantation.

[35] DeFilippis EM (2020). Challenges in Heart Transplantation in the Era of COVID-19. Circulation.

[36] Arabi M, Majdalani M, El Hajj MA, Nemer G, Sawaya F, Obeid M, Bitar FF (2011). The status of pediatric cardiology at a tertiary center in Lebanon. J Med Liban.

